# Determination of the Dielectrophoretic Force Induced by the Photovoltaic Effect on Lithium Niobate

**DOI:** 10.3390/mi13020316

**Published:** 2022-02-18

**Authors:** Alessio Meggiolaro, Sebastian Cremaschini, Davide Ferraro, Annamaria Zaltron, Mattia Carneri, Matteo Pierno, Cinzia Sada, Giampaolo Mistura

**Affiliations:** Department of Physics and Astronomy, University of Padua, Via Marzolo 8, 35131 Padua, Italy; alessio.meggiolaro@studenti.unipd.it (A.M.); sebastian.cremaschini@studenti.unipd.it (S.C.); davide.ferraro@unipd.it (D.F.); annamaria.zaltron@unipd.it (A.Z.); mattia.carneri@unipd.it (M.C.); matteo.pierno@unipd.it (M.P.); cinzia.sada@unipd.it (C.S.)

**Keywords:** optofluidics, dielectrophoresis, lithium niobate, photovoltaic effect, pendant droplet

## Abstract

The actuation of droplets on a surface is extremely relevant for microfluidic applications. In recent years, various methodologies have been used. A promising solution relies on iron-doped lithium niobate crystals that, when illuminated, generate an evanescent electric field in the surrounding space due to the photovoltaic effect. This field can be successfully exploited to control the motion of water droplets. Here, we present an experimental method to determine the attractive force exerted by the evanescent field. It consists of the analysis of the elongation of a pendant droplet and its detachment from the suspending syringe needle, caused by the illumination of an iron-doped lithium niobate crystal. We show that this interaction resembles that obtained by applying a voltage between the needle and a metallic substrate, and a quantitative investigation of these two types of actuation yields similar results. Pendant droplet tensiometry is then demonstrated to offer a simple solution for quickly mapping out the force at different distances from the crystal, generated by the photovoltaic effect and its temporal evolution, providing important quantitative data for the design and characterization of optofluidic devices based on lithium niobate crystals.

## 1. Introduction

The wetting of solid surfaces can be modified by changing material or surface properties, such as surface chemistry or micro- or nanoscale topography [1,2], or by introducing additional stimuli, including electric [3,4] and magnetic [5,6] fields, light [7,8], temperature gradients [9] and acoustic vibrations [10,11]. This is relevant for many different applications, from microfluidics to energy harvesting [12,13]. In recent years, electrowetting on dielectric (EWOD) has been shown to be one of the most versatile and effective methods to actively control the wetting of a solid surface by a droplet [14]. In this approach, a metallic electrode is coated with a hydrophobic dielectric layer and a water droplet is used as the other electrode to create a capacitive structure, but with an electrode contact area that depends on the extent to which the dielectric layer is wetted [4]. As a result of this effect, the contact angle decreases with the applied voltage, which can be used to actuate water droplets. Electrowetting has found extensive applications, thanks to its fast response time and the large forces achievable, from the millimeter to micrometer scale [14,15]. However, it requires the realization of electrodes and their cumbersome connection to voltage suppliers. A promising alternative to the presence of metallic electrodes is based on the photovoltaic effect exhibited inside certain ferroelectrics, such as lithium niobate, LiNbO_3_ [16,17,18]. Upon appropriate illumination, an electric field is generated within the material with a strength that can be as high as 200 kV/cm and photoinduced charges are redistributed on the surface [19]. The photoinduced electric field E extends outside the active optical material (evanescent field) and can be exploited to manipulate neutral micro- and nano-objects through dielectrophoretic forces $F_{DEP}$ [20,21,22,23]. In general, the dielectrophoretic force arises from the interaction between a non-uniform electric field and a neutral, dielectric body and can be approximated as $F_{\mathrm{DEP}}=-\nabla \left(p\cdot E\right)\sim -\nabla E^{2}$, where **p** is the induced polarization [21,22,23,24,25,26]. In the case of a homogeneous spherical particle of radius r and dielectric constant $\epsilon_{p}$, immersed in a lossless dielectric fluid of dielectric constant $\epsilon_{m}$, this force is given by the following simple equation [23,27]:

(1) $$F_{DEP}=2\pi \varepsilon \epsilon_{m}r^{3}K\nabla E^{2}$$

where K is the real part of the Clausius–Mossotti factor $\left[\epsilon_{p}\left(\omega \right)-\epsilon_{m}\left(\omega \right)\right]/\left[\epsilon_{p}\left(\omega \right)+2\epsilon_{m}\left(\omega \right)\right],$ $\;\epsilon_{p}\left(\omega \right)$ and $\epsilon_{m}\left(\omega \right)$ being the complex dielectric constants of the particle and of the surrounding fluid, respectively, calculated at the pulsation ω of the electric field [28]. If we assume a spherical water droplet, immersed in air, the droplet will always be attracted towards regions of high field strength.

Recently, the photovoltaic effect has been exploited to manipulate water droplets [29,30,31,32,33]. In fact, unlike EWOD systems that consider a fixed configuration of microfabricated electrodes, the photoinduced electric pattern can be easily redesigned on the same Fe:LiNbO_3_ by discharging the substrate. Despite the large diffusion from this approach, during the last few years, the magnitude of the dielectrophoretic forces used to drive the droplets over the Fe:LiNbO_3_ surfaces have not yet been measured. Here, we propose a simple technique that allows one to evaluate the dielectrophoretic force acting on a water droplet. It is a variation of the pendant droplet tensiometry, commonly used to measure the surface tension of a liquid [34,35]. A water droplet of known volume is attached to the tip of the stainless steel needle of a vertically held syringe. The dielectrophoretic force $F_{DEP}$ is derived by evaluating the curvature of the elongated droplet. We have applied this technique to measure the dielectrophoretic force acting on a water droplet due to the interaction with an electric field, generated in the following two distinct ways: (i) by a voltage applied between the metallic surface (E–DEP) and the needle, and (ii) with the evanescent field due to the photovoltaic effect of a Fe:LiNbO_3_ crystal (P–DEP). Measurement of the forces derived in the two configurations yields consistent results, confirming the validity and flexibility of this approach.

This paper is organized as follows: in the next section, we briefly describe the materials and experimental setups; we present the data analysis procedure and discuss the results obtained from the E–DEP and P–DEP measurements.

## 2. Materials and Methods

### 2.1. Fe-Doped Lithium Niobate Samples

In this work, a z-cut iron-doped lithium niobate (Fe:LiNbO_3_) crystal is used, having a diameter of 3 inches and a thickness of 1 mm. The crystal is supplied by PI-KEM Limited and presents a uniform iron concentration of 0.1%mol (18.8 × 10^18^ at/cm^3^), ensuring a rapid photorefractive response. The concentration of donor ions Fe^2+^ is obtained by optical absorption measurement using a spectrophotometer (Jasco V-670) in the range of 300–2000 nm [36] and is (4.6 ± 0.1) × 10^18^ at/cm^3^. This value implies a reduction degree *R*, defined as the ratio between the number of donors Fe^2+^ and acceptors Fe^3+^, of 0.32 ± 0.01.

### 2.2. Dielectrophoretic Force on a Water Droplet Generated by Photovoltaic and Electrostatic Charges

**Figure 1:**
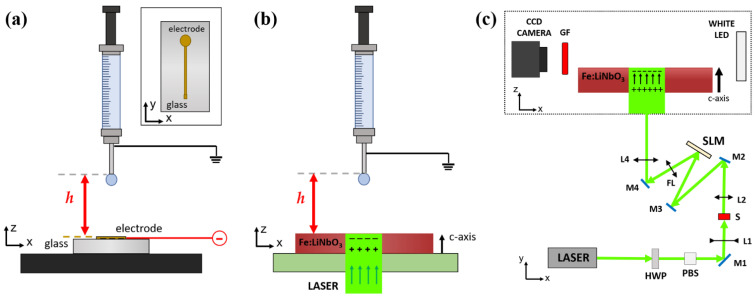
(**a**) Experimental setup for the E–DEP evaluation. A glass slide coated with a gold electrode is connected to a voltage generator. The top view of the electrode is reported in the right-side box. (**b**) Experimental setup for P–DEP evaluation. Laser light illuminates the Fe:LiNbO_3_ crystal and creates a distribution of charges of opposite sign on the two main surfaces. (**c**) Schematic view of the optical path. A CCD camera and a white LED backlight are aligned to allow for a lateral view of the experimental area. The laser beam is expanded with lenses L1, L2 to illuminate the spatial light modulator (SLM) area. The desired light pattern is set and projected on the crystal by the Fourier lens (FL). Mirrors M1–M4 are used to guide the beam and bring it up to the vertical plane (*x,z*). The power of the beam is tuned by a half-wave plate (HWP) coupled with a polarizer beam splitter (PBS). A mechanical shutter (S) is used to control the illumination time. In both setups, a syringe pump provides the formation of a pendant droplet of known volume.

In this work, two distinct experimental setups have been used, both with the objective of evaluating the effect produced by surface charge accumulation on a droplet suspended above it, as shown in Figure 1. In the first setup, see Figure 1a, electrostatic charges are created on the surface of a conductive substrate, while in the second setup, see Figure 1b, charges are generated on the main faces of Fe:LiNbO_3_ crystals through the photovoltaic effect.

The experimental setup designed to generate an electrostatic field below the pendant droplet and to evaluate the resulting dielectrophoretic force (E–DEP) is shown in Figure 1a. Ultrapure water droplets (resistivity 18.2 MΩ·cm) of known volume (Ω = 2.7 μL) are generated with a computer-controlled syringe pump (PHD 22/2000, Harvard apparatus) equipped with a glass syringe (1 mL, SGE) that mounts a stainless steel needle with an outer diameter of 0.2 mm. The droplet remains attached to the needle due to capillarity, assuming the conventional pendant droplet shape [34,35,37]. A CCD camera (Basler acA1300–200 μm) equipped with a telecentric objective is used to collect the lateral profile of the droplet, while a white LED provides back illumination. Below the needle, a glass slide is coated with a gold electrode deposited by magnetron-sputtering having a keyhole shape. In detail, the latter is formed by a circular 4 mm diameter spot connected to a 0.2 × 50 mm^2^ stripe (see inset in Figure 1a). The center of the circular part of the electrode is aligned with the needle of the syringe placed above. A high-voltage power supply provides a tunable voltage to the gold electrode up to 2000 V, while the needle is grounded. During a typical experiment, the distance *h* between the needle and the electrode is fixed, and the voltage is progressively increased until the droplet falls.

The setup to evaluate the dielectrophoretic force due to the photovoltaic effect (P–DEP) is schematically shown in Figure 1b,c. It presents the same components as the previously presented experimental setup for pendant droplet generation and imaging. The Fe:LiNbO_3_ crystal is placed on a three-axes motorized sample holder (M126.CG Linear Slide Translational Stage, PI) designed to allow illumination from the bottom. The light pattern projected on the sample is achieved by a linearly polarized laser beam (Class 4, Azur Light Systems, power 1 W max, *λ* = 532 nm, Pessac, France). The gaussian beam emitted by the laser is modulated in phase by a spatial light modulator (Pluto-NIR-011, Holoeye Photonics, Berlin, Germany) to project on the Fe:LiNbO_3_ crystal a circular spot having uniform light intensity and diameter *d* = 4 mm. The uniform light spot generated by the spatial light modulator yields a charged area with sharper contours than in the case of a gaussian beam, which would produce an expanding charge distribution with exposure time [24]. A properly tilted mirror ensures that the incident light beam illuminates the Fe:LiNbO_3_ crystal perpendicularly to its main z-cut surfaces and that the circular spot is aligned with the needle placed above. The intensity of the light pattern on the sample can be tuned between 0 and *I* = 11.1 kW/m^2^ using a half-wave plate (HWP) coupled with a polarizer beam splitter (PBS). An emission filter (GF, MF620-52, Thorlabs, Newton, NJ, USA) is placed in front of the objective to remove any residual reflection of the laser beam on the droplet. In analogy to the electrostatic case, during a typical experiment, the distance *h* between the needle and the upper Fe:LiNbO_3_ surface is fixed; then the laser light is turned on and the time required to induce the droplet to fall is measured. After each measurement run, the crystal is fully discharged by immersing it in water for about 20 min.

## 3. Results and Discussion

### 3.1. Dielectrophoretic Force on Pendant Droplet: Electrostatic Effect (E–DEP)

In a typical pendant droplet configuration, the droplet formed at the tip of the needle remains suspended because the capillary force $F_{c}$ acting along the contact line balances the droplet weight $F_{g}$. The maximum capillary force $F_{c,max}$ exerted on a needle of external radius R can be approximated as follows [38]:

(2) $$F_{c,max}=2\pi R\gamma \psi$$

where γ is the surface tension of the droplet liquid and ψ is a correction factor depending on the droplet volume Ω. In our case, we used water droplets of Ω = 2.7 ± 0.1 µL, resulting in ψ=0.93, with a measured surface tension γ=72±1 mJ/m^2^. Since the needle has radius R = 0.1 mm, Formula (2) yields $F_{c,max}=41.8\pm 0.8\;$µN. If a charged electrode is placed underneath, the water droplet, assumed to be a pure dielectric with no free charges, is attracted downward due to the dielectrophoretic force $F_{DEP}$.

**Figure 2:**
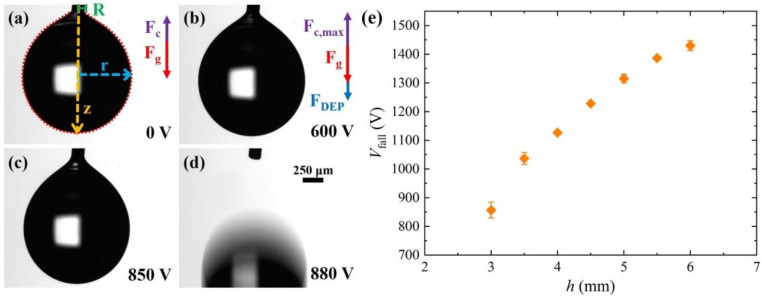
(**a**–**d**) Sequence of snapshots showing the shape of a pendant droplet at increasing voltages applied to the underlying electrode. The graphical insets indicate the main geometric quantities and the forces involved. The distance of the needle tip from the electrode is *h* = 3 mm and the volume of the droplet is 2.7 μL. (**e**) Variation of the minimum voltage *V*_fall_ required to detach the droplet from the needle with distance *h*. If not visible, the error bars are the size of the symbols.

Figure 2a shows the characteristic geometry of a water droplet suspended to the needle in the absence of an electric field. The distance of the needle tip from the electrode is *h =* 3 mm. As a result of the pull action of the weight $F_{g}$, the shape of the droplet is not spherical, but rather an elongated pear-like shape. When a voltage is applied to the electrode, the droplet becomes more elongated due to the contribution of the dielectrophoretic force $F_{DEP}$, which is parallel to the direction of $F_{g}$, as shown in Figure 2b. As the voltage is increased, the deformation becomes slightly more pronounced, see Figure 2c, until at *V*_fall_ = 880 V, the droplet detaches from the needle and falls, see Figure 2d and Video S1. This means that at *V*_fall_, the pull action of the dielectrophoretic force and the droplet weight overcome the maximum capillary force exerted by the needle and, thus, we get the following equation:

(3) $$F_{DEP}=F_{c,max}-F_{g}=15\pm 1.3\;µN$$

The results of the voltage *V*_fall_ as a function of height *h* are plotted in the graph of Figure 2e, where each data point is the mean of at least three repeated measurements and the error bars represent the corresponding standard deviations. As expected, *V*_fall_ increases with *h* because, for a given voltage, the electric field experienced by the droplet decays with the distance from the electrode.

An alternative method to estimate the dielectrophoretic force is to analyze the curvature of the contour of a pendant droplet. When the droplet is at equilibrium, the hydrostatic pressure jump across the interface equalizes the Laplace pressure for each height *z* along the droplet [37], seen below:

(4) $$\rho g^{*}z=\gamma C$$

where ρ = 997 kg/m^3^ is the water density at *T* = 25 °C, $g^{*}$ is the effective gravitational acceleration [39] and *C* is the curvature at the surface. Because the dielectrophoretic force acts in the same direction as gravity, the total pull experienced by the droplet can be expressed as follows:

(5) $$F_{g}+F_{DEP}=\varrho Vg+F_{DEP}=\varrho Vg\left(1+\frac{F_{DEP}}{\varrho Vg}\right)\equiv \varrho Vg^{*}$$

In other words, the effective gravitational acceleration $g^{*}=\varrho Vg\left(1+\frac{F_{DEP}}{\varrho Vg}\right)$ quantifies the dielectrophoretic force; in the absence of an electric field, $g^{*}=g$, otherwise $g^{*}>g$. At each point *r*(*z*) of the surface, as shown in Figure 1a, the curvature can be parametrized in cylindrical coordinates, as follows [29]:

(6) $$C=-\frac{\frac{d^{2}r}{dz^{2}}}{{\left(1+{\left(\frac{dr}{dz}\right)}^{2}\right)}^{\frac{3}{2}}}+\frac{1}{r{\left(1+{\left(\frac{dr}{dz}\right)}^{2}\right)}^{1/2}}$$

The droplet profile can be fitted numerically [40], according to Equations (2)–(4), to extract $g^{*}$, given ρ and *γ*. Thus, we have applied this procedure to the droplet profiles extracted from the video frames, right before the droplet fall. In this way, it is possible to estimate the value of $F_{DEP}$ required to detach the water droplet. Table 1 shows the results obtained for *h ≥* 3 mm; for shorter distances, the droplet touches the surface during elongation. All $F_{E\text{–}DEP}$ values agree within one standard deviation and their mean value ${\bar{F}}_{E\text{–}DEP}=14\pm 2$ µN is perfectly consistent with the value of 15.1 ± 1.3 µN, deduced above.

### 3.2. Dielectrophoretic Force on the Pendant Droplet: Photovoltaic Effect (P–DEP)

**Figure 3:**
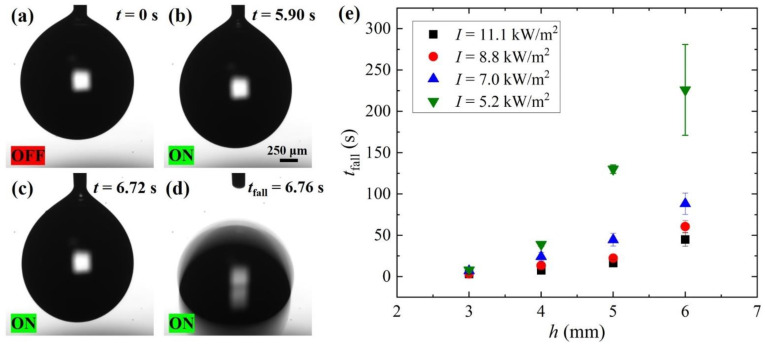
(**a**–**d**) Sequence of snapshots showing the detachment of a pendant droplet caused by the charges generated on the surface of the underlying Fe:LiNbO_3_ crystal illuminated with an intensity *I* = 7.0 kW/m^2^. The distance of the needle tip from the crystal is *h* = 3 mm and the volume of the droplet is 2.7 μL. (**e**) Variation of the time *t*_fall_ required to detach the droplet from the needle with distance *h* and for different light intensities *I*.

An analogous behavior is observed illuminating the Fe:LiNbO_3_ crystal, as shown in Figure 3 for *h* = 3 mm and Video S2. Initially, the laser is off and the droplet is deformed due to the balance between the capillary and gravity forces, see Figure 3a. When the light is turned on at *t* = 0 s, charges are expected to accumulate progressively on the Fe:LiNbO_3_ surfaces in the illuminated area [16] and, as a consequence, the droplet elongates due to the dielectrophoretic force, as shown in Figure 3b. During illumination, this elongation progressively increases, see Figure 3c, until at a time *t*_fall_ = 6.76 s, the droplet detaches from the needle and falls down, see Figure 3d. This evolution resembles that discussed in Figure 2a–d, observed by increasing the applied voltage. The graph in Figure 3e shows the variation of the characteristic time *t*_fall_ with the distance *h* between 3 and 6 mm and for different light intensities; each data point is the mean of at least three measurements, repeated under the same conditions, and the error bars represent the corresponding standard deviations. The light intensities were chosen to yield *t*_fall_ sufficiently low to significantly limit the evaporation of the droplets during the measurements. For a given illumination intensity *I*, *t*_fall_ increases with *h*, particularly for *I* = 5.2 kW/m^2^, while for a fixed *h*, the time required to detach the droplet decreases with increasing *I.* These results can be rationalized by taking into account the temporal evolution of the light-induced electric field within the lithium niobate crystal [16,17], as follows:

(7) $$E_{in}=E_{sat}\left(1-e^{-t/\tau }\right)$$

where the amplitude $E_{sat}$ is the highest electric field achievable within the crystal and is proportional to the amount of Fe^3+^. The parameter *τ* represents the photovoltaic time constant and is defined as τ=k/(R·I), where *k* a is constant typical of the material [16,17], *R* = [Fe^2+^]/[Fe^3+^] the reduction degree. Therefore, because the electric field experienced by the droplet decays with the distance from the crystal, at higher values of *h*, the photoinduced charge density required to detach the droplet must increase, and this implies, for a given *I*, longer illumination times. Similarly, using higher *I* leads to faster charge accumulation and, thus, given a certain *h*, *t*_fall_ decreases with increasing *I*, see Figure 3e.

The value of $F_{P\text{–}DEP}$, generated by the charges photoinduced on the Fe:LiNbO_3_ crystal, is deduced from the analysis of the droplet contour immediately before the droplet falls, as done in the previous section, to compare it to the force computed in Equation (3). As reported in Table 1, for *I* = 8.8 kW/m^2^, all $F_{P\text{–}DEP}$, the values are rather similar, confirming that the force necessary to overcome the capillary force is always the same at any distance *h*, although the required illumination time *t*_fall_ differs by more than one order of magnitude. Importantly, their mean value ${\bar{F}}_{P\text{–}DEP}=13.3\pm 1.5$ μN in the case of the photovoltaic effect is compatible with the value ${\bar{F}}_{E\text{–}DEP}=14\pm 2$ μN found in the electrostatic experiment. This confirms the analogy between the two experiments and the capability of the pendant droplet to work as a probe for the dielectrophoretic force. 

### 3.3. Dynamics of the Dielectrophoretic Force by the Photovoltaic Effect (P–DEP)

**Figure 4:**
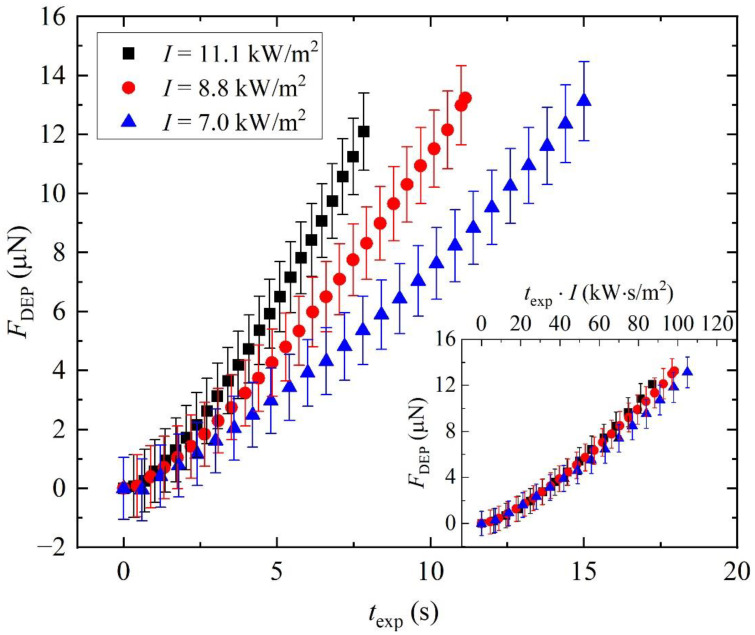
Variation of the dielectrophoretic force $F_{P\text{–}DEP}$ induced by the photovoltaic effect over the exposure time $t_{\exp}$ of the Fe:LiNbO_3_ acting on a pendant water droplet for different intensities *I*. The inset shows the same data plotted as a function of the light exposure rate $t_{\exp}\cdot I$. The distance of the needle tip from the crystal is fixed at *h* = 4 mm and the volume of the droplet is 2.7 μL.

The pendant droplet method can also be applied to investigate time-dependent processes, such as charge accumulation on Fe:LiNbO_3_ surfaces. As an example, Figure 4 shows the temporal evolution of the $F_{P\text{–}DEP}$ acting on the droplet attached to the needle, distanced *h* = 4 mm from the top surface of the Fe:LiNbO_3_ crystal. The force is plotted against the exposure time *t*_exp_ of the crystal to different light intensities. It is determined by analyzing each video frame acquired during the illumination. The final points of each curve correspond to the frame at which the droplet falls. As expected, this event is independent of the beam intensity and yields similar $F_{P\text{–}DEP}$ values, between 12.1 μN and 13.2 μN, in very good agreement, within one standard deviation, with those listed in Table 1. The increase over time of $F_{P\text{–}DEP}$ reflects the increase in the evanescent field, due to the photovoltaic effect predicted by Equation (6). More precisely, Equation (1) indicates that the temporal evolution of $F_{P\text{–}DEP}\left(t\right)$ is the same as that of $E_{in}{\left(t\right)}^{2}$. Since the characteristic time constant *τ* is inversely proportional to the light intensity, this explains why $F_{P\text{–}DEP}$ grows more slowly at lower *I*. Furthermore, if we plot the same data in terms of the so-called light exposure rate, equal to $t_{\exp}\cdot I$, [24] we expect from Equation (6) that all the data collapse on the same master curve, which depends only on material properties of the Fe:LiNbO_3_ crystal. The graph inset confirms the validity of this scaling.

## 4. Conclusions

We have described a simple technique to measure the dielectrophoretic force acting on a water droplet due to an electric field. It consists of a variation of the pendant droplet technique, commonly employed to derive the surface tension of a liquid. We have successfully applied it to probe the forces generated in standard electrowetting (electric field due to the voltage generated by a power supply) and optowetting (electric field due to the illumination of a lithium niobate crystal) applications. It does not require sophisticated set-ups, can be easily applied to different experimental situations and provides reliable spatial and temporal resolutions. We feel that this pendant droplet technique can yield quantitative information relevant for the design and characterization of microfluidic devices, using electric fields for the droplet actuation.

## Figures and Tables

**Table 1:** The dielectrophoretic force required to detach the droplet from the needle evaluated at different heights *h* from the E–DEP and P–DEP experiments. The values of $F_{P\text{–}DEP}$ refer to a light intensity *I* = 8.8 kW/m^2^ and a volume of the droplet Ω = 2.7 μL.

h (mm)	$F_{E\text{–}DEP}$ (μN)	$F_{P\text{–}DEP}$ (μN)
3.0 ± 0.1	14.9 ± 1.5	14.2 ± 1.5
4.0 ± 0.1	15.8 ± 1.5	12.0 ± 1.4
5.0 ± 0.1	12.2 ± 1.4	12.0 ± 1.4
6.0 ± 0.1	11.9 ± 1.4	15.0 ± 1.5
${\bar{F}}_{DEP}$	14 ± 2	13.3 ± 1.5

## Data Availability

Data are contained within the article or Supplementary Materials.

## Supplementary Materials

The following are available online at https://www.mdpi.com/article/10.3390/mi13020316/s1, Video S1: Detachment of a pendant water droplet due to the electric field induced by the voltage applied by the E–DEP experimental setup. Video S2: Detachment of a pendant water droplet due to the illumination of an iron-doped lithium niobate crystal by the P–DEP experimental setup.
